# NMR characterization of HIV-1 reverse transcriptase binding to various non-nucleoside reverse transcriptase inhibitors with different activities

**DOI:** 10.1038/srep15806

**Published:** 2015-10-29

**Authors:** Ratsupa Thammaporn, Maho Yagi-Utsumi, Takumi Yamaguchi, Pornthip Boonsri, Patchreenart Saparpakorn, Kiattawee Choowongkomon, Supanna Techasakul, Koichi Kato, Supa Hannongbua

**Affiliations:** 1 Department of Chemistry, Faculty of Science, Kasetsart University, Chatuchak, Bangkok, Thailand; 2 Institute for Molecular Science and Okazaki Institute for Integrative Bioscience, National Institutes of Natural Sciences, Japan; 3 Graduate School of Pharmaceutical Sciences, Nagoya City University, Japan; 4 Department of Chemistry, Faculty of Science, Srinakharinwirot University, Bangkok, Thailand; 5 Center of Nanotechnology, Kasetsart University, Chatuchak, Bangkok, Thailand; 6 Department of Biochemistry, Faculty of Science, Kasetsart University, Chatuchak, Bangkok, Thailand

## Abstract

Human immunodeficiency virus type 1 reverse transcriptase (HIV-1 RT) is an important target for antiviral therapy against acquired immunodeficiency syndrome. However, the efficiency of available drugs is impaired most typically by drug-resistance mutations in this enzyme. In this study, we applied a nuclear magnetic resonance (NMR) spectroscopic technique to the characterization of the binding of HIV-1 RT to various non-nucleoside reverse transcriptase inhibitors (NNRTIs) with different activities, i.e., nevirapine, delavirdine, efavirenz, dapivirine, etravirine, and rilpivirine. ^1^H-^13^C heteronuclear single-quantum coherence (HSQC) spectral data of HIV-1 RT, in which the methionine methyl groups of the p66 subunit were selectively labeled with ^13^C, were collected in the presence and absence of these NNRTIs. We found that the methyl ^13^C chemical shifts of the M230 resonance of HIV-1 RT bound to these drugs exhibited a high correlation with their anti-HIV-1 RT activities. This methionine residue is located in proximity to the NNRTI-binding pocket but not directly involved in drug interactions and serves as a conformational probe, indicating that the open conformation of HIV-1 RT was more populated with NNRTIs with higher inhibitory activities. Thus, the NMR approach offers a useful tool to screen for novel NNRTIs in developing anti-HIV drugs.

Human immunodeficiency virus type 1 reverse transcriptase (HIV-1 RT) plays an important role in HIV-1 replication by catalyzing the conversion of single-stranded RNA into double-stranded DNA. This enzyme is one of the most promising targets for anti-HIV drug development to suppress the production of new viral particles. The structure of HIV-1 RT consists of an asymmetric heterodimer of two subunits, a 66 kDa subunit (p66) containing both polymerase and RNase H domains, and a 51 kDa subunit (p51) containing only a polymerase domain^1^,^2^,^3^. Each polymerase domain is comprised of four subdomains: fingers, thumb, palm, and connection^1^,^3^. The p66 subunit carries the functional sites including the polymerase active site, the RNase H domain and the non-nucleoside binding site, whereas p51 provides the structural foundation^4^.

HIV-1 RT inhibitors can be divided into two classes, nucleoside reverse transcriptase inhibitors (NRTIs) and non-nucleoside reverse transcriptase inhibitors (NNRTIs). NRTIs are nucleoside analogs lacking the 3′-OH group and acts as a chain terminator of DNA synthesis. NNRTIs are small molecules that bind to a hydrophobic pocket located in proximity to the polymerase active site on the p66 subunit^5^,^6^. It is expected that NNRTIs are able to circumvent the toxic side effects associated with nucleoside chain termination^7^. Accordingly, the NNRTI binding pocket is considered to be an important target for further development of novel anti-HIV-1 drugs. Five NNRTIs, nevirapine, delavirdine, efavirenz, etravirine, and rilpivirine, have currently been approved by the U.S. Food and Drug Administration^8^. However, the efficiencies of these inhibitors are impaired by mutations in HIV-1 RT^9^, requiring continuous development of novel NNRTIs capable of inhibiting both wild-type and mutated HIV-1 RT enzymes. Hence, a detailed knowledge about the interactions between this enzyme and NNRTIs in solution is crucial for antiviral therapy against acquired immunodeficiency syndrome.

Biophysical and structural approaches are useful for rapid, efficient development of small molecule inhibitors targeting HIV-1 RT. X-ray crystallography offers atomic images of the different binding modes of HIV-1 RT between NRTIs and NNRTIs^5^,^6^,^8^,^10^,^11^,^12^,^13^. The availability of these crystallographic structures has greatly facilitated the optimization of NNRTIs. Nuclear magnetic resonance (NMR) is also a useful method for studying HIV-1 RT binding to drugs. Although applying the NMR technique to analysis of large proteins remains challenging, this spectroscopic method provides valuable information regarding dynamic aspects of ligand binding. It has been reported that selective isotope labeling with ^13^C at the methyl side chain of methionine offers useful spectroscopic probes for investigating the structures and dynamics of larger proteins^14^,^15^,^16^,^17^,^18^. Zheng *et al.* previously reported heteronuclear single-quantum coherence (HSQC) spectra for observing signals from the methionine methyl groups of the HIV-1 RT p66 subunit in the absence and presence of nevirapine, with assignments based on the site-directed mutagenesis method^16^,^17^. In this study, the response of HIV-1 RT binding to its ligands in solution was probed with methyl ^13^C resonances.

In the present study, we have applied the NMR technique to characterize the interactions of HIV-1 RT with various NNRTIs with different inhibitory activities, nevirapine, delavirdine, efavirenz, dapivirine, etravirine, and rilpivirine (Fig. 1). We found that the methyl ^13^C chemical shift of M230 in the p66 subunit, which is located in close proximity to the inhibitor binding pocket, serves as a useful indicator of the efficacy of these NNRTIs.

## Results and Discussion

### Spectral assignments of the apo form of HIV-1 RT with the ^13^C-labeled p66 subunit

In the present NMR study, HIV-1 RT complex composed of ^13^C-labeled p66 and unlabeled p51 was prepared by bacterial expression using [methyl-^13^C]methionine. The recombinant p66 subunit possesses six intrinsic methionine residues and an extra methionine residue at its N-terminus. The ^1^H-^13^C HSQC spectrum of the apo form of the ^13^C-labeled HIV-1 RT protein gave four peaks (supplementary Fig. S1). To assign each methyl resonance, six different mutants of HIV-1 RT were prepared, substituting each methionine in the p66 subunit with leucine^16^,^17^. The ^1^H-^13^C HSQC spectra of these mutants were compared with those of the wild type, thereby identifying peaks originating from M16, M184, and M357, because these peaks were missing in the spectra of the corresponding mutants (supplementary Fig. S1). The remaining mutants, i.e., M41L, M164L, and M230L, exhibited virtually identical ^1^H-^13^C HSQC spectra with those of the wild type, indicating that these methionine residues gave no observable peaks, presumably because their side chains are buried in the protein and thereby have low mobility. These observations are consistent with the ^1^H-^13^C HSQC spectral data reported previously^16^,^17^. We confirmed that the sharp peak (indicated by an asterisk) was derived from the N-terminal extra methionine because it was eliminated by treatment with methionine aminopeptidase (supplementary Fig. S2).

### Spectral changes upon drug binding to HIV-1 RT

**Figure 2:**
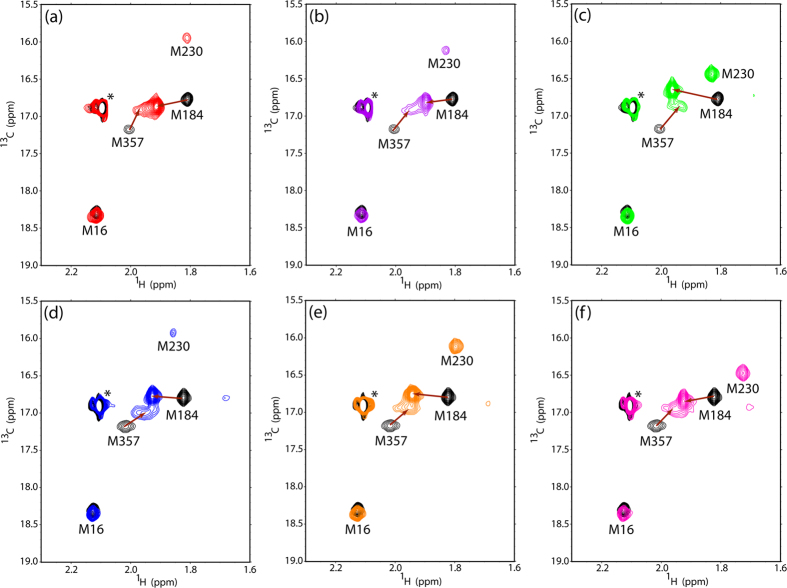
Superposition of the ^1^H- ^13^C HSQC spectra of apo HIV-1 RT (black) and HIV-1 RT complexed with (a) nevirapine, (b) efavirenz, (c) rilpivirine, (d) delavirdine, (e) dapivirine, and (f) etravirine.

To examine the effects of NNRTIs bound to HIV-1 RT, ^1^H-^13^C HSQC spectral data of HIV-1 RT with the [methyl-^13^C]methionine-labeled p66 subunit were collected in the presence of six NNRTIs, nevirapine, delavirdine, efavirenz, dapivirine, etravirine, and rilpivirine, as shown in Fig. 2. Chemical shift changes of the methionine methyl resonances were observed upon the addition of these NNRTIs to HIV-1 RT. NNRTIs examined induced significant chemical shift changes for the resonances originating from M184 and M357. Moreover, the M230 peak became observable upon addition of these drugs. The chemical shift of the M16 resonance was little affected by drug binding, whereas the M41 and M164 resonances remained unobservable.

The crystal structures of the apo form without a ligand and the complexed form with these inhibitors suggest that the binding of NNRTIs stabilizes the open conformation of HIV-1 RT. The thumb and fingers domains are more distant than in the closed conformation adopted by the apo form of HIV-1 RT, in which the thumb domain is in contact with the fingers domain with occlusion of the NNRTI-binding pocket^1^,^19^,^20^,^21^. M184 and M230 are located in the vicinity of this pocket but not directly involved in drug binding. In the drug-binding open conformation, M230 is more exposed to the solvent, in contrast to the apo closed conformation in which this methionine residue gave no observable peak because of its low mobility. Therefore, this methionine signal might serve as a conformational probe for characterizing NNRTI-binding to HIV-1 RT.

**Figure 3:**
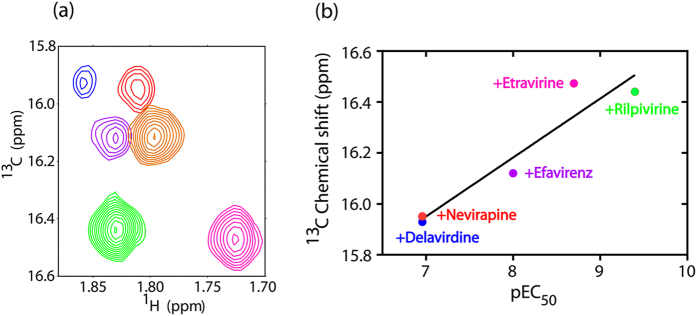
(**a**) Superposition of ^1^H-^13^C HSQC spectra showing the M230 peak of the p66 subunit in HIV-1 RT with nevirapine (red), efavirenz (purple) and rilpivirine (green), delavirdine (blue), dapivirine (orange), and etravirine (magenta). (**b**) Correlation between anti-HIV-1 RT activity reported by Yang *et al.*^22^ and the ^13^C chemical shift of M230 of the HIV-1 RT p66 subunit upon binding to these inhibitors. Linear regression analyses showed a significant correlation between the chemical shift and pEC_50_ values (*r*^*2*^ = 0.91, *p* = 0.012, *p < 0.05).

Spectral comparison among the six NNRTI-bound forms of HIV-1 RT indicated significant chemical variation of the M230 peak (Fig. 3a). The methyl ^13^C chemical shift of M230 exhibited a high correlation with anti-HIV-1 RT activity reported by Yang *et al.*^22^ (*r*^*2*^ = 0.91 and *p* = 0.012, *p < 0.05), as shown in Fig. 3b, demonstrating its utility for evaluating and even predicting the efficacies of NNRTIs.

The ^13^C chemical shift of the methionine methyl group can refer to side chain conformations, associated with χ^3^ values: the ^13^C chemical shifts of 16 ppm and 19 ppm correspond to *gauche* and *trans* conformations, respectively, whereas 17 ppm indicates flexibly exchanging *trans* and *gauche* conformations^23^. In the closed apo structure of HIV-1 RT (PDB codes 1DLO and 3DLK), M230 is buried, exclusively exhibiting the *gauche* conformation, while it occasionally adapted a *trans* conformation in the rilpivirine-bound open form (PDB code 3MEE), presumably with increased mobility. Under this circumstance, ^13^C chemical shift of M230 reflects drug-induced population shifts of the open and closed conformations. The intensity of the M230 resonance in the rilpivirine- and etravirine-bound forms of HIV-1 RT was higher than that in the nevirapine-, delavirdine- and efavirenz-bound forms. This indicates that diarylpyrimidine-based inhibitors (DAPYs) such as rilpivirine and etravirine fix the open conformation most tightly, rendering M230 exposed with its highest mobility (with a partial *trans* conformation) in comparison with nevirapine, delavirdine, and efavirenz. In other words, fixation in open conformation by nevirapine, delavirdine, and efavirenz are not complete. Consistent with this interpretation, the enzyme bound to efavirenz remains under dynamic equilibrium between the open and closed forms^24^. On the basis of these data, we conclude that the open conformation of HIV-1 RT was more populated in complexes with NNRTIs with higher inhibitory activities.

In summary, we applied NMR spectroscopy to probe NNRTI binding to HIV-1 RT using the methionine methyl ^13^C resonances from the p66 subunit. We confirmed that the compounds examined in this study share the same binding pocket. Moreover, our NMR data revealed that the methyl ^13^C chemical shift of M230 can be used as an indicator of the efficacy of NNRTI, offering a useful tool in screening for novel inhibitors in developing anti-HIV drugs.

## Materials and Methods

### Materials

Powdered rilpivirine, delavirdine, dapivirine, and etravirine were purchased from MedChem Express. Nevirapine and efavirenz were synthesized by Dr. Supanna Techasakul (Department of Chemistry, Faculty of Science, Kasetsart University).

### Preparation of HIV-1 RT variants by site-directed mutagenesis

Site-directed mutagenesis was performed to prepare six variants of the p66 subunit of HIV-1 RT, M16L, M41L, M164L, M184L, M230L, and M357L. The recombinant plasmids containing HIV-1 RT genes, pGEX3X 25 for p66 and pET33B^26^ for p51, were previously constructed. The pGEX3X vector carrying the wild-type p66 subunit of HIV-1 RT was used as a template for site-directed mutagenesis. PCR was performed with two synthetic oligonucleotide primers containing the desired mutation using the KOD-plus DNA polymerase (TOYOBO). PCR products were digested by *DpnI* (TOYOBO) and transformed into the *Escherichia coli* strain DH5α. The mutations were confirmed by DNA sequencing using an ABI 3130*xl* genetic analyzer (Applied Biosystems).

### Protein expression and purification of wild-type and mutated HIV-1 RT

Wild-type and mutated p66 subunit proteins were individually expressed in the *E. coli* BL21(DE3)-RIL strain (Agilent Technologies) in M9 medium containing 50 μg/ml of ampicillin with L-[methyl-^13^C]methionine (Cambridge Isotope Laboratories) using previously described protocols^27^,^28^. The wild-type p51 subunit was expressed separately using M9 medium with 15 μg/ml of kanamycin. Production of recombinant proteins was induced by the addition of 0.5 mM isopropyl β-_D_-thiogalactopyranoside at 16 °C for 16–18 h. The harvested cells of p51 and p66 HIV-1 RT were suspended and mixed into 50 mM Tris-HCl (pH 7.5) containing 0.5 mM EDTA, 50 mM NaCl, 1 mM DTT, 5% (w/v) glycerol, 0.5% (w/v) Triton X-100, and protease inhibitor cocktail (Sigma-Aldrich) and then disrupted by sonication. The HIV-1 RT protein was sequentially purified from cell lysates with a DEAE cellulose column (Whatman), a phosphocellulose P11 column (Whatman), a Chelating Sepharose Fast Flow (GE Healthcare) charged with nickel sulfate, and a RESOURCE S column (GE Healthcare). Finally, the HIV-1 RT protein was purified by Superdex-200 (HiLoad 16/60) gel filtration using a FPLC column (GE Healthcare). All purification steps of HIV-1 RT were performed at 4 °C with buffer containing protease inhibitors.

To confirm the existence of the extra N-terminal methionine, HIV-1 RT protein was incubated with methionine aminopeptidase (Clontech) at 37 °C for 12 h, and checked by NMR.

### NMR measurements

All NMR measurements were made using an AVANCE800 (Bruker BioSpin) spectrometer equipped with a cryogenic probe. The probe temperature was set at 25 °C. The wild-type or mutated HIV-1 RT was dissolved at a concentration of 27 μM in 10 mM Tris-HCl-d_11_ (pD 7.6) containing 200 mM KCl, 1.5 mM sodium azide, and 4 mM MgCl_2_. A 20 mM stock solution of each inhibitor was prepared in dimethylsulfoxide-d_6_ and was added in five-fold molar excess to the protein. Spectra were processed and analyzed with the programs Topspin and SPARKY^29^.

## Additional Information

**How to cite this article**: Thammaporn, R. *et al.* NMR characterization of HIV-1 reverse transcriptase binding to various non-nucleoside reverse transcriptase inhibitors with different activities. *Sci. Rep.* **5**, 15806; doi: 10.1038/srep15806 (2015).

## Supplementary Material

Supplementary Information

## Figures and Tables

**Figure 1:**
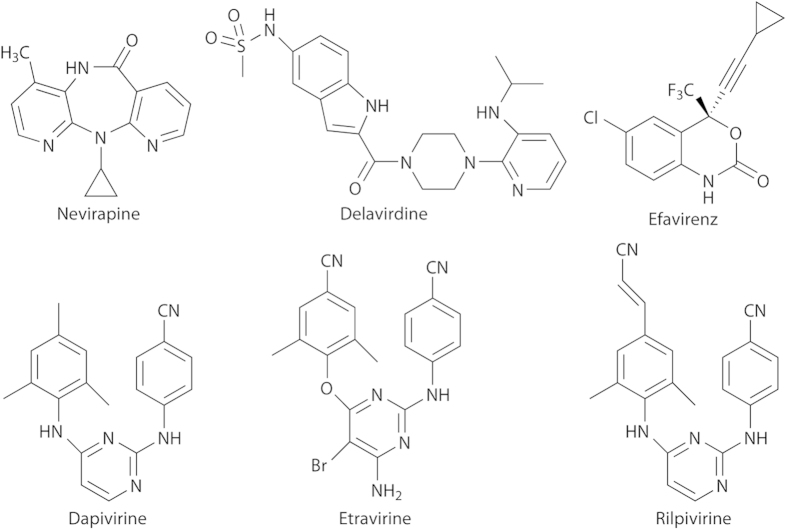
Structures of nevirapine, delavirdine, efavirenz, dapivirine, etravirine, and rilpivirine.
