# Supplementary material for: NMR characterization of HIV-1 reverse transcriptase binding to various non-nucleoside reverse transcriptase inhibitors with different activities
Source: Sci Rep. 2015 Oct 29;5:15806. doi: 10.1038/srep15806 (PMC4625163; doi:10.1038/srep15806)
Supplement: Supplementary Information [file srep15806-s1.pdf]

Supplementary Information

NMR characterization of HIV-1 reverse transcriptase binding to various non-nucleoside reverse transcriptase inhibitors with different activities

Ratsupa Thammaporn[1,2], Maho Yagi-Utsumi[2,3], Takumi Yamaguchi[2,3], Pornthip Boonsri[4], Patchreenart Saparpakorn[1,5], Kiattawee Choowongkomon[6], Supanna Techasakul[1], Koichi Kato[2,3*], and Supa Hannongbua[1,5]*

[1]*Department of Chemistry, Faculty of Science, Kasetsart University, Chatuchak, Bangkok Thailand*

[2]*Institute for Molecular Science and Okazaki Institute for Integrative Bioscience, National Institutes of Natural Sciences, Japan*

[3]*Graduate School of Pharmaceutical Sciences, Nagoya City University, Japan*

[4]*Department of Chemistry, Faculty of Science, Srinakharinwirot University, Bangkok Thailand*

[5]*Center of Nanotechnology, Kasetsart University, Chatuchak, Bangkok Thailand*

[6]*Department of Biochemistry, Faculty of Science, Kasetsart University, Chatuchak, Bangkok Thailand*

*Corresponding author: fscisph@ku.ac.th and kkato@phar.nagoya-cu.ac.jp

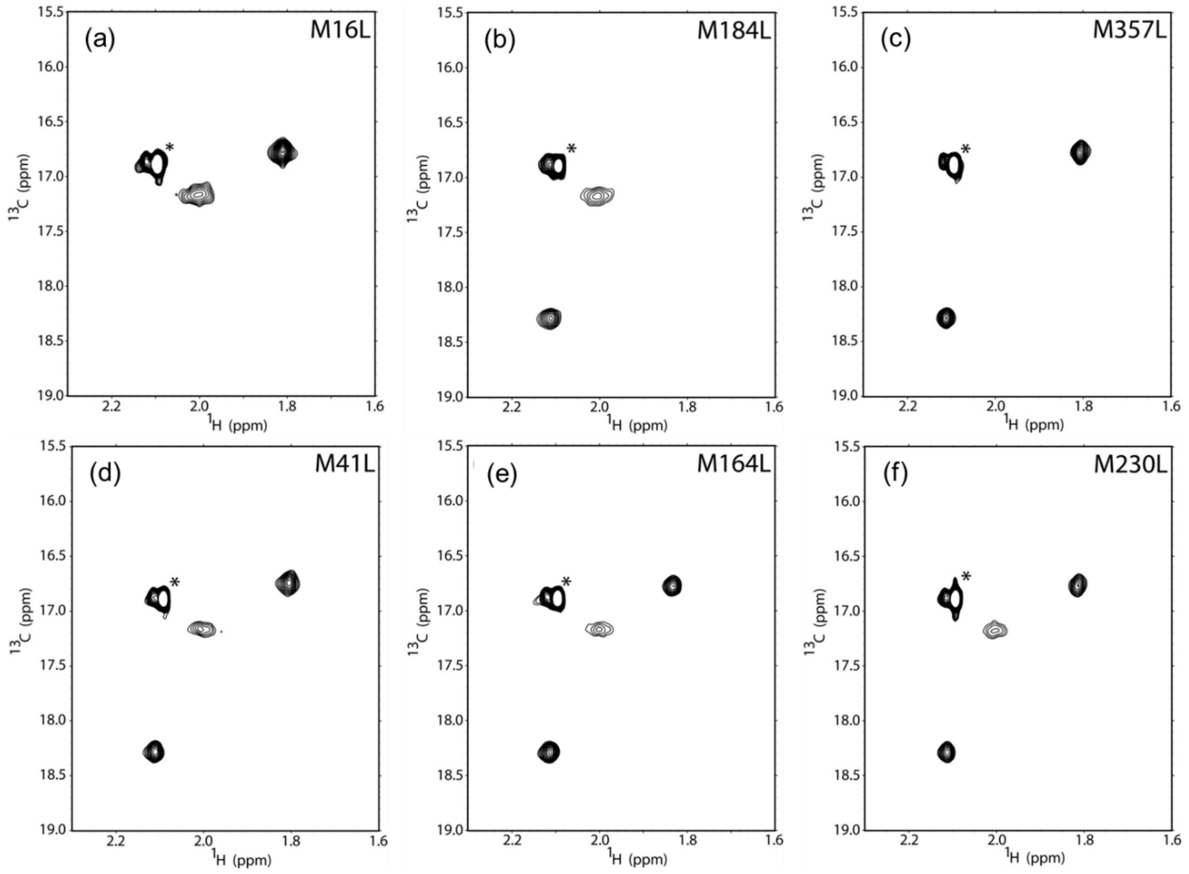

Supplementary Fig. S1. ^{1}H-^{13}C HSQC spectra of the [methyl-^{13}C]methionine-labeled p66 mutants of HIV-1 RT: (a) M16L, (b) M184L (c) M357L (d) M41L, (e) M164L, and (f) M230L. The resonance originating from the N-terminal extra methionine is marked with an asterisk.

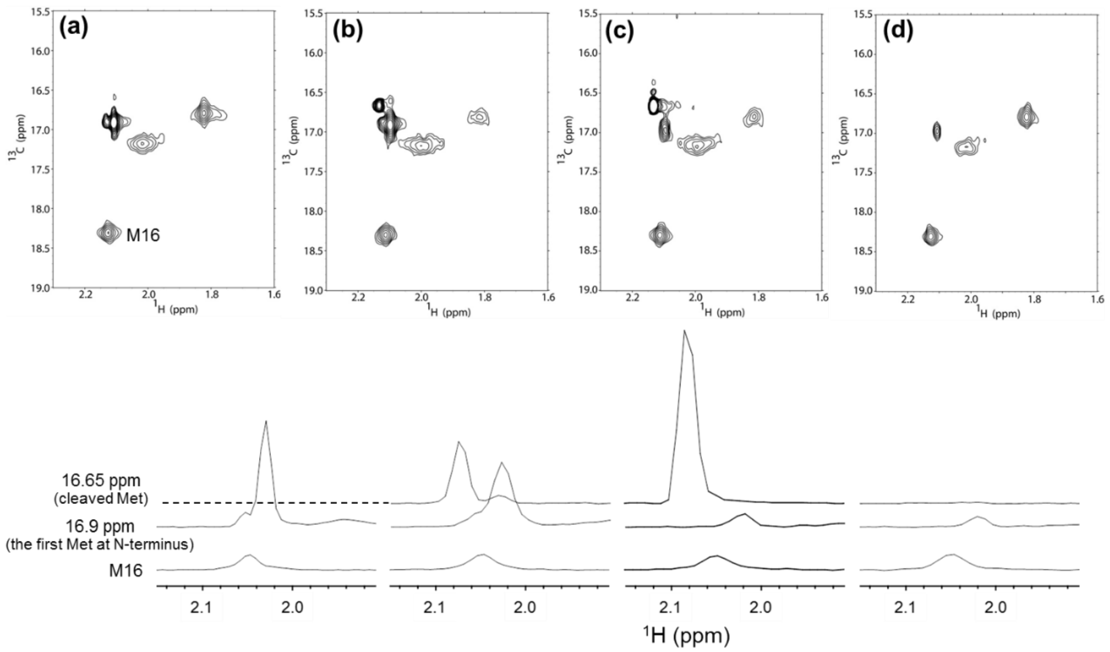

Supplementary Fig. S2. ^{1}H-^{13}C HSQC spectra of HIV-1 RT before (a) and after incubation in the presence of methionine aminopeptidase (Clontech) at 70:1 unit of HIV-1 RT: aminopeptidase at 37°C for 1.5 h (b) and 12 h (c). (d) The ^{1}H-^{13}C HSQC spectrum of the same sample after removing the cleaved methionine using a PD-10 desalting column (GE Healthcare). 1D slices across the ω_1 dimension of HSQC spectra at 16.9 ppm (corresponding to the N-terminal first methionine) and at 16.65 ppm (corresponding to liberated methionine) were normalized by the intensity of the peak of M16.
